# Further investigations on the influence of protein phosphatases on the signaling of muscarinic receptors in the atria of mouse hearts

**DOI:** 10.1007/s00210-024-02973-4

**Published:** 2024-02-03

**Authors:** Ulrich Gergs, Silke Wackerhagen, Tobias Fuhrmann, Inka Schäfer, Joachim Neumann

**Affiliations:** Institut Für Pharmakologie Und Toxikologie, Medizinische Fakultät, Martin-Luther-Universität Halle-Wittenberg, D-06097, Magdeburger Str. 4, 06112 Halle, Germany

**Keywords:** Protein phosphorylation, PP2A, PP1, Inhibitor-2, Muscarinic receptor, Transgenic mice, Heart

## Abstract

The vagal regulation of cardiac function involves acetylcholine (ACh) receptor activation followed by negative chronotropic and negative as well as positive inotropic effects. The resulting signaling pathways may include G_i/o_ protein-coupled reduction in adenylyl cyclase (AC) activity, direct G_i/o_ protein-coupled activation of ACh-activated potassium current (I_KACh_), inhibition of L-type calcium ion channels, and/or the activation of protein phosphatases. Here, we studied the role of the protein phosphatases 1 (PP1) and 2A (PP2A) for muscarinic receptor signaling in isolated atrial preparations of transgenic mice with cardiomyocyte-specific overexpression of either the catalytic subunit of PP2A (PP2A-TG) or the inhibitor-2 (I2) of PP1 (I2-TG) or in double transgenic mice overexpressing both PP2A and I2 (DT). In mouse left atrial preparations, carbachol (CCh), cumulatively applied (1 nM–10 µM), exerted at low concentrations a negative inotropic effect followed by a positive inotropic effect at higher concentrations. This biphasic effect was noted with CCh alone as well as when CCh was added after β-adrenergic pre-stimulation with isoprenaline (1 µM). Whereas the response to stimulation of β-adrenoceptors or adenosine receptors (used as controls) was changed in PP2A-TG, the response to CCh was unaffected in atrial preparations from all transgenic models studied here. Therefore, the present data tentatively indicate that neither PP2A nor PP1, but possibly other protein phosphatases, is involved in the muscarinic receptor-induced inotropic and chronotropic effects in the mouse heart.

## Introduction

Acetylcholine (ACh) binds to muscarinic ACh receptors expressed in the sarcolemma of cardiomyocytes. The G_i_-coupled M_2_ ACh receptor is the main muscarinic ACh receptor in the mammalian heart, expressed in the atrium and ventricle as well as in the conduction system including the sinoatrial (SA) node and atrioventricular (AV) node (overview in Saw et al. (2018)). Effects of M_2_ receptor stimulation in the heart are hyperpolarization in the SA node and a slowed spontaneous depolarization resulting in reduced sinus rate, a shortened action potential duration and probably as a result a decreased contractile force in the atrium, and a decreased conduction velocity in the AV node (Dhein et al. 2001).

Recent studies have demonstrated that muscarinic ACh receptor activation in sinoatrial nodal cells leads to a decrease in beating rate either via reduced activation of the hyperpolarization-activated current (I_f_) or L-type Ca^2+^ current by a G_i_-mediated reduction in adenylyl cyclase (AC) activity or via direct G_i_-coupled activation of ACh-activated potassium current (I_K.ACh_) (Lyashkov et al. 2009).

In left atrial preparations of wild-type mice, carbachol (CCh), an ACh analogue, induced a transient negative inotropic effect followed by a positive inotropic response (Kitazawa et al. 2009). Using antagonists or muscarinic M_2_ or M_3_ receptor single knockout mice or M_2_ and M_3_ muscarinic receptor double knockout mice, the authors demonstrated that the negative inotropic effect of CCh was M_2_ receptor–mediated and the positive inotropic effect of CCh was M_3_ receptor–mediated (Kitazawa et al. 2009).

In guinea pig cardiac preparations (isolated perfused heart or isolated ventricular cardiomyocytes), acetylcholine decreased the isoprenaline-stimulated phosphorylation of phospholamban (PLB) and troponin inhibitor (TnI), but without reducing cAMP levels or the protein kinase A activity ratio (Gupta et al. 1994). The addition of the protein phosphatase inhibitor okadaic acid blocked the ACh-mediated effects on protein phosphorylation. It was suggested by the authors that ACh reduced protein phosphorylation independently of cAMP (Gupta et al. 1994). Later, the data were expanded to inotropic effects in guinea pig papillary muscles. Carbachol reduced the positive inotropic effect of isoprenaline and these effects were blocked by inhibition of phosphatases. Therefore, it was suggested that the ventricular cardiac effects of muscarine receptor stimulation involve protein phosphatase activation (Neumann et al. 1995; Neumann and Scholz 1998). Similar results were reported in canine ventricular preparations where cantharidin attenuated the carbachol-induced negative inotropic effect (Chu et al. 2003).

Here, we tested the hypothesis that protein phosphatases are involved in the cardiac response to muscarinic receptor stimulation. Since it is currently not known which protein phosphatases are involved, we started to test this hypothesis by studying the inotropic and chronotropic effects in isolated atrial preparations of PP2A-transgenic mice (PP2A-TG), PP1-inhibitor-2 mice (I2-TG), double transgenic mice (PP2A-TGxI2-TG = DT), and as control wild-type mice (WT). In I2-TG mice, we noted not only a reduced cardiac PP1 activity, but also a reduced PP2A activity (Krause et al. 2018). This effect has been neglected up to now. Here, we generated the double transgenic model with reduced cardiac PP1 activity (like in I2-TG), but increased PP2A activity (like in PP2A-TG). For the experimental setup, our choice fell on atrial preparations, firstly because carbachol is effective under both basal and adrenergically stimulated conditions and secondly because in isolated right atrial preparations, the effect on the sinus node can be elegantly examined. Since muscarinic receptor stimulation usually, in mice and man, results in concentration- and time-dependent negative and positive inotropic effects in cardiac preparations, we compared the response to muscarinic receptor stimulation with the response to β-adrenoceptor stimulation and A_1_-adenosine receptor stimulation.

## Materials and methods

### Transgenic mice

For this study, transgenic mice with cardiomyocyte-specific overexpression either of the catalytic subunit of PP2A (PP2A-TG) (Gergs et al. 2004) or of the inhibitor-2 of PP1 (I2-TG) (Krause et al. 2018) were used. Some experiments were performed with double transgenic mice (PP2A-TGxI2-TG = DT) obtained by crossbreeding PP2A-TG with I2-TG mice. Transgene-positive mice (CD1 background) were routinely identified by PCR assay of ear punch genomic DNA. On the protein level, the overexpression of the catalytic subunit of PP2A was 2.5-fold in PP2A-TG hearts (Gergs et al. 2004) and the overexpression of I2^PP1^ was ninefold in I2-TG hearts (Krause et al. 2018). The phenotypes of the mouse models have been described before (Cheng et al. 2010; Gergs et al. 2004, 2022; Hoehn et al. 2015; Wijnker et al. 2011). Briefly, the life span and fertility of the monotransgenic mice were unchanged compared to WT, and the life span of DT mice (monitored up to the date of the experiment) was also unchanged compared to WT. PP2A-TG mice develop age-dependently cardiac hypertrophy, decreased cardiac function, and diminished response to β-adrenergic stimulation (Gergs et al. 2004). The phenotype of I2-TG mice is unremarkable compared to WT (Krause et al. 2018). For the experiments, 6 to 8-month-old mice of each gender were used (we did not detect any difference between cardiac preparations from male or female mice during this study). The investigation conforms to the Guide for the Care and Use of Laboratory Animals published by the National Research Council (US) 2011 (National Academies Press (US) 2011). Animals were maintained and handled according to approved protocols of the animal welfare committees of the Martin-Luther University of Halle-Wittenberg, Germany. All efforts were made to minimize suffering.

### Mouse atrial preparations

A force of contraction of mouse left and right atrial preparations was recorded under isometric conditions as often described by our group (Gergs et al. 2013, 2017, 2019b, 2021; Neumann et al. 2003). In brief, mice were euthanatized by i. p. injection of pentobarbital sodium (50 mg kg^−1^). Then, hearts were excised and the right and left atria were isolated and mounted in 10-ml organ baths containing a Tyrodes’s solution composed of 119.8 mM NaCl 5.4 mM KCl, 1.8 mM CaCl_2_, 1.05 mM MgCl_2_, 0.42 mM NaH_2_PO_4_, 22.6 mM NaHCO_3_, 0.05 mM Na_2_EDTA, 0.28 mM ascorbic acid, and 5.05 mM glucose. The bathing solution was continuously gassed with 95% O_2_ and 5% CO_2_ to stabilize the pH 7.4 and maintained at 37 °C. A force of contraction (FOC) was measured with inductive force transducers connected to a bridge amplifier and digitizer (PowerLab system, ADInstruments, Oxford, UK). Each atrial preparation was stretched to the length of its individual maximal FOC. The left atrial preparations were electrically stimulated at 1 Hz (field stimulation) with rectangular pulses of 5 ms duration and a voltage that was ~ 10–20% over the threshold of initiation of contraction. The right atrial preparations were prepared with an intact sinus node to maintain spontaneous beating and were used to study chronotropic effects. Before contraction experiments with carbachol were started, adenosine deaminase (1 µg·ml^−1^ for 30 min) was added to avoid any interferences of residual or released adenosine (Boknik et al. 2009). From the recorded force of contraction, the first derivate (dF/dt) and the time parameters of single contractions (left atrial preparations) and the beating rate (right atrial preparations) were calculated using the chart software (ADInstruments, Oxford, UK).

This biphasic response to CCh was analyzed by calculation of the relative change in the force of contraction for each CCh concentration normalized to the individual baseline force before the effect begins as described earlier (Froldi et al. 1994; Gergs et al. 2008). That means in detail: the “pre-drug value” for the negative inotropic effect of the concentration *X*_n_ is the positive inotropic effect of the concentration before (*X*_n-1_). Correspondingly, the “pre-drug value” for the positive inotropic effect of the concentration *X*_n_ is the negative inotropic effect of the very same concentration (*X*_n_). This procedure was performed for all concentrations *X*_n_(*n* = 10^−9^ to 10^−5^). These negative and positive relative changes in force were added, respectively, to get a concentration–response curve and expressed in %.

### Protein phosphatase assays

Protein phosphatase activity was measured with [^32^P]-phosphorylase *a* as substrate as described before (Gergs et al. 2004). Complete frozen heart samples were pulverized in liquid nitrogen. Then, the tissue powder was homogenized in a homogenization buffer containing 20 mM Tris–HCl (pH 7.4), 5 mM EDTA, 2 mM EGTA, 0.1% (v/v) β-mercaptoethanol, 0.5 mM phenylmethylsulfonyl fluoride, and 1 mM benzamidine three times for 30 s each with a Polytron PT-10 (Kinematica, Luzern, Switzerland) at 4 °C. Finally, the homogenate was cleared by centrifugation (10 min; 14,000 × *g*; 4 °C) and the protein concentration of the supernatant was measured using a Bradford assay. The homogenates were diluted to 0.5 mg protein/ml in 50 mM Tris–HCl (pH 7.4) and aliquots of 10 µl (containing 5 µg of protein) were continued to be used. To differentiate between PP1 and PP2A activity, 10 nM okadaic acid (buffer only for the total activity) was added to the half of samples and preincubated for 10 min at 30 °C. Twenty microliters of a reaction mixture containing the [^32^P]-labeled substrate and 2.5 mM Tris–HCl (pH 7.4), 12.5 mM caffeine, 0.25 mM EDTA, and 0.25% (v/v) β-mercaptoethanol were added to the preincubated homogenates. After 30 min at 30 °C, the reaction was terminated by adding 20 µl of 50% trichloroacetic acid on ice together with 30 µl of 20 mg/ml bovine serum albumin. The precipitated proteins were sedimented by centrifugation (14,000 × *g*; 5 min; 4 °C) and an aliquot of the supernatants with the released phosphate was counted in a liquid scintillation counter. The samples including 10 nM okadaic acid were representative mainly for the PP1 activity. The PP2A activity was calculated by: total activity – activity with okadaic acid.

### Reagents

(-)-Isoprenaline ( +)-bitartrate, carbachol, and R-PIA (R(-)-N(6)-(2)-phenylisopropyl adenosine) were purchased from Sigma-Aldrich (Munich, Germany). All other chemicals used were of the highest purity grade commercially available and demineralized water was used throughout the experiments.

### Statistics

All data are presented as means ± SEM. The statistical analyses were performed either by the Student’s *t*-test if appropriate or by the analysis of variance (ANOVA) followed by Tukey’s multiple comparisons test for more than two variables. For small sample groups (*n* < 6), a non-parametric test was chosen. A *p*-value < 0.05 was set as the statistical significance level. For statistical calculations and graphical presentations, the software GraphPad Prism 9.0 (GraphPad Software, San Diego, CA, USA) was used.

## Results

We carried out contraction experiments under isometric conditions in isolated atrial preparations from WT, PP2A-TG, I2-TG, and DT mice. The left atria were field-stimulated at 1 Hz to analyze the force of contraction and the spontaneously beating right atria were used to analyze the beating rate, more specifically to analyze the effects on the sinus node.

### Effects of isoprenaline

**Fig. 1:**
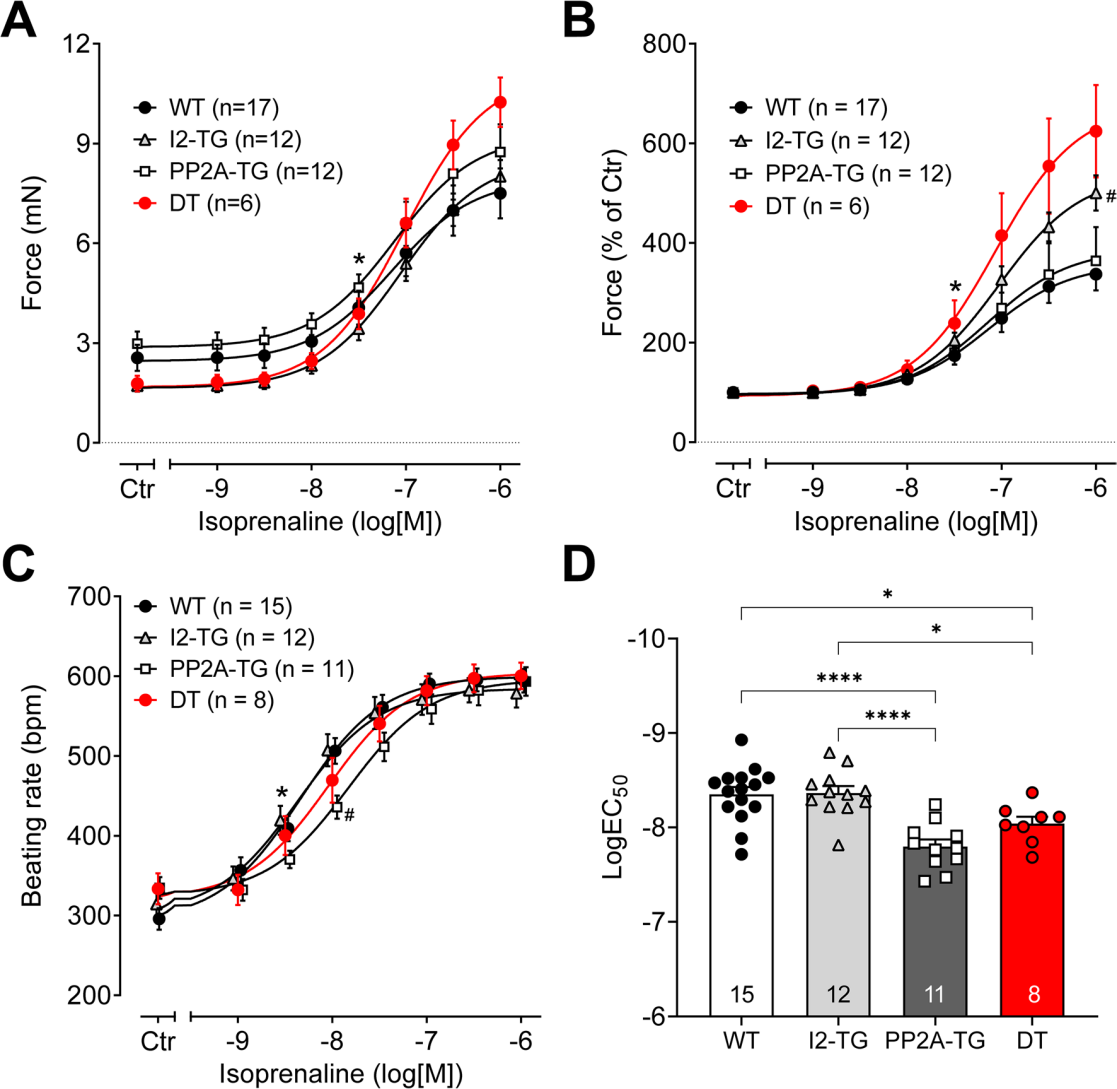
Summarized concentration–response curves for the effect of isoprenaline on force of contraction in electrically driven (1 Hz) left atrial preparations (**A**, **B**) and on the beating rate in right atrial preparations (**C**, **D**) from PP2A-TG, I2-TG, DT, and WT. Numbers in brackets give the number of experiments. Ctr is the pre-drug value. **A** Force of contraction in milli Newton (mN) and **B** shows the same data in % of control (Ctr) to visualize the efficiency of isoprenaline. *First *p* < 0.05 vs. Ctr; ^#^*p* < 0.05 vs. WT. **C** Beating rate in beats per minute (bpm). *First *p* < 0.05 vs. Ctr; ^#^*p* < 0.05 vs. WT and I2-TG. **D** The logarithm of the EC_50_ values demonstrates the potency of isoprenaline to increase the beating rate in right atrial preparations. Significant differences (*p* < 0.05) are indicated by brackets

In isolated left atrial preparations, cumulatively applied isoprenaline (1 nM – 1 µM) only increased the force of contraction. The potency of isoprenaline to induce a positive inotropic effect was comparable in all genotypes tested (-logEC_50_: 7.03–7.14) (Fig. 1A, B). Whereas the potency of isoprenaline was not different, the efficacy of isoprenaline was increased in I2-TG and DT left atrial preparations (Fig. 1A, B). In spontaneously beating right atrial preparations, the effects were different: cumulatively applied isoprenaline (1 nM–1 µM) increased the beating rate from about 300 bpm to the same maximum (about 600 bpm) in all genotypes (Fig. 1C), but the potency of isoprenaline to induce a positive chronotropic effect was reduced clearly in PP2A-TG and to a lesser extent in DT (Fig. 1D).

### Effects of carbachol

**Fig. 2:**
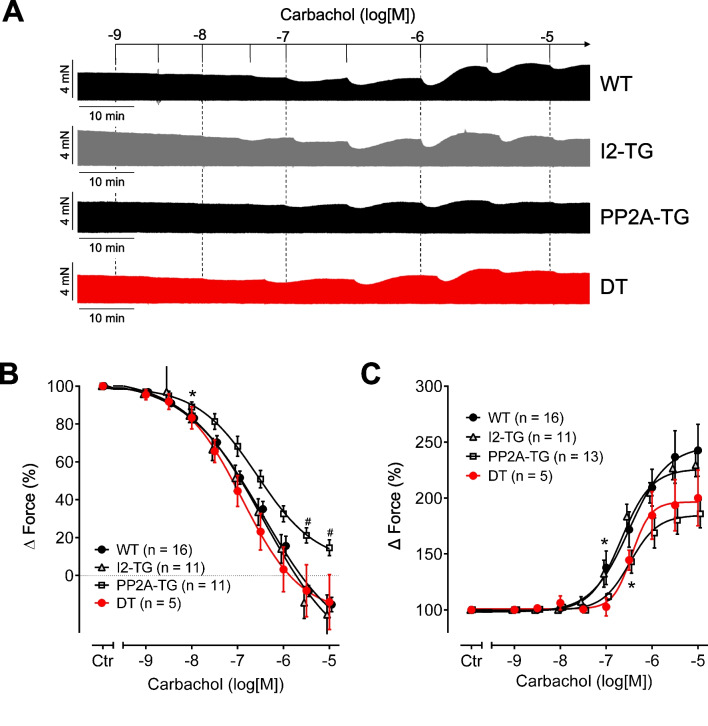
Concentration–response curves for the effect of carbachol on force of contraction in electrically driven (1 Hz) left atrial preparations from PP2A-TG, I2-TG, DT, and WT. Numbers in brackets give the number of experiments. Ctr is the value before carbachol application. **A** Original recordings demonstrate the biphasic effect of carbachol. Ordinates: force of contraction (mN); horizontal bars: time (min). **B** Only the negative inotropic effect of carbachol is plotted and **C** only the positive inotropic effect of carbachol is plotted as delta milli Newton in % of pre-drug value, respectively, as described in the method section. *First *p* < 0.05 vs. Ctr; ^#^*p* < 0.05 vs. WT and I2-TG

In mouse left atrial preparations, cumulatively applied CCh (1 nM–10 µM) exerted a negative inotropic effect followed by a positive inotropic effect at higher concentrations (Fig. 2A). Moreover, even at a given low concentration of CCh, there was an initial fall in force of contraction followed over time by an increase in force of contraction that usually did not surpass pre-drug value. This biphasic response makes it difficult to independently quantitate the negative and positive inotropic effects of CCh. To dissect the differences, we calculated the relative change in the force of contraction for each CCh concentration normalized to the individual baseline force before the effect begins as others and we ourselves have done in previous studies (Froldi et al. 1994; Gergs et al. 2008). The negative and positive relative changes in force were added, respectively, to get a concentration–response curve. A drawback of this method is that both negative and positive inotropic effects were underestimated, but the primary aim was to compare the response to CCh in different genotypes rather than to measure the exact inotropic response.

In the first set of experiments, CCh was cumulatively applied alone (that means without any pre-stimulation). The potency of CCh for the negative (-logEC_50_: 6.4–6.9) or positive (-logEC_50_: 6.5–6.7) inotropic effect in left atrial preparations was not different between genotypes (Fig. 2B, C). But the maximum response to CCh was reduced in left atrial preparations from PP2A-TG compared to WT and I2-TG (*p* < 0.05 for the negative inotropic effect; not significant for the positive inotropic effect).

**Fig. 3:**
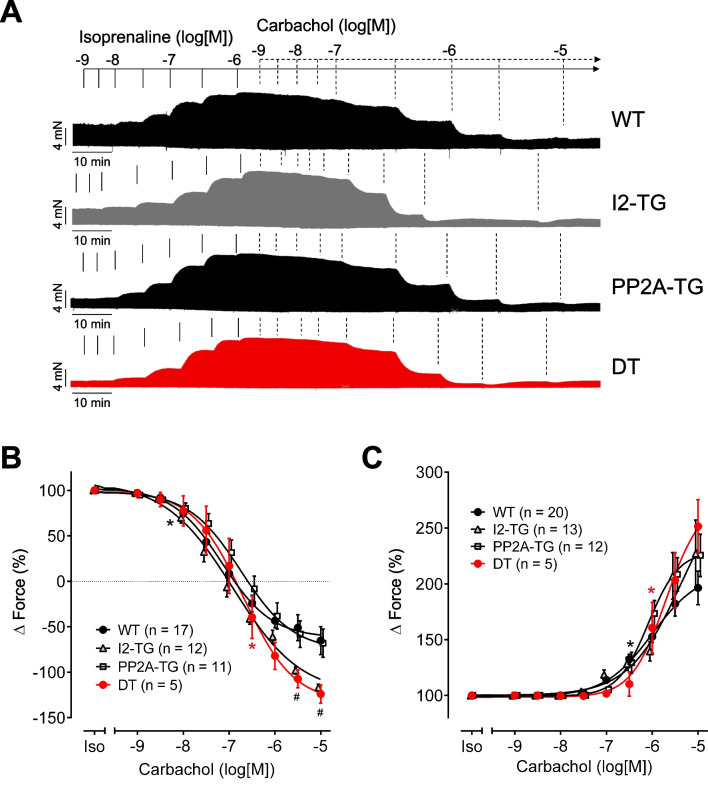
Concentration–response curves for the effect of isoprenaline followed by carbachol on force of contraction in electrically driven (1 Hz) left atrial preparations from PP2A-TG, I2-TG, DT, and WT. Numbers in brackets give the number of experiments. Iso indicates the maximum response to isoprenaline (1 µM) and was used here as control for the carbachol application. **A** Original recordings demonstrate that the biphasic effect of carbachol is still present, but shifted to higher concentrations. Ordinates: force of contraction (mN); horizontal bars: time (min). **B** Only the negative inotropic effect of carbachol after pre-stimulation with Iso is plotted and **C** only the positive inotropic effect of carbachol after pre-stimulation with Iso is plotted as delta milli Newton in % of pre-drug value, respectively, as described in the method section. *First *p* < 0.05 vs. Iso; ^#^*p* < 0.05 vs. WT and PP2A-TG

Next, the atrial preparations were pre-stimulated by 1 µM isoprenaline before CCh was applied. Also in this setting, CCh had a biphasic effect in left atrial preparations (Fig. 3A). Again, the potency of CCh for the negative (-logEC_50_: 6.4–6.9) and probably for the positive (EC_50_: not calculable because of a missing plateau at the end of the concentration–response curve) inotropic effect was not different between genotypes (Fig. 3B, C). The maximum negative inotropic response to CCh was increased in left atrial preparations from I2-TG and DT compared to WT and PP2A-TG (Fig. 3B).

**Fig. 4:**
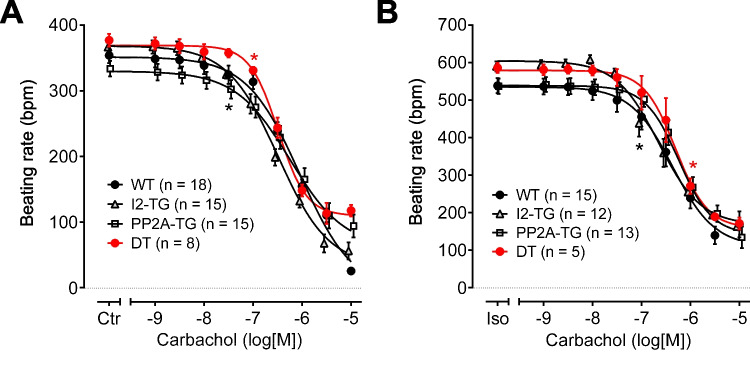
Summarized concentration–response curves for the effect of carbachol alone (**A**) or after pre-stimulation with 1 µM isoprenaline (Iso) (**B**) on the beating rate in right atrial preparations from PP2A-TG, I2-TG, DT, and WT are shown. Numbers in brackets give the number of experiments. Ctr is the pre-drug value. Iso indicates the maximum response to isoprenaline and was used here as control for the carbachol application. *First *p* < 0.05 vs. Ctr or Iso

In right atrial preparations, CCh alone exerted a negative chronotropic effect that was not different between the genotypes (-logEC_50_: 6.0–6.5) (Fig. 4A). Similar results were found in isoprenaline-stimulated right atrial preparations, where the negative chronotropic effect of CCh was again not different between the genotypes (-logEC_50_: 6.2–6.7) (Fig. 4B).

### Effects of R-PIA

**Fig. 5:**
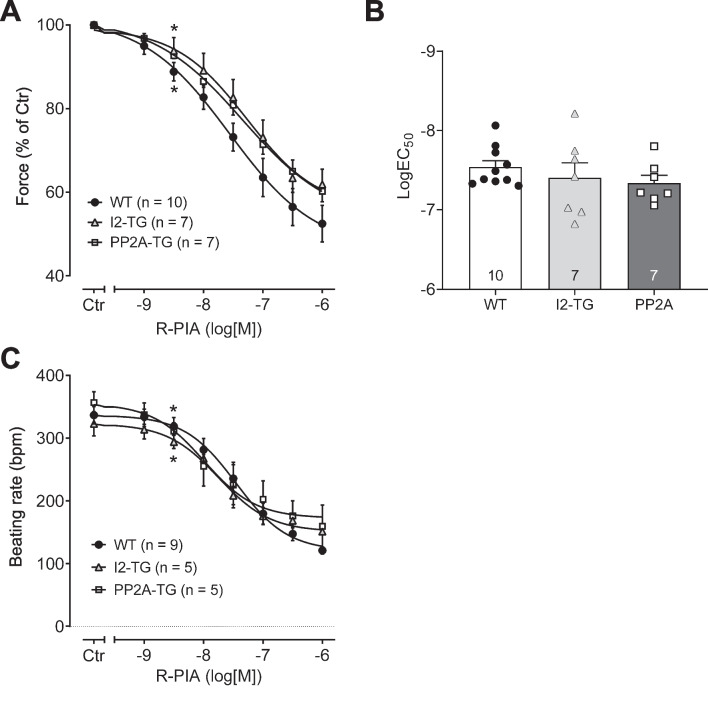
Summarized concentration–response curves for the negative inotropic and chronotropic effect of the adenosine receptor agonist R-PIA in electrically driven (1 Hz) left atrial preparations (**A**, **B**) and in right atrial preparations (**C**), respectively, from PP2A-TG, I2-TG, and WT. Numbers in brackets give the number of experiments. Ctr is the value before R-PIA application. **A** Force of contraction in % of control (Ctr). **B** The logarithm of the EC_50_ values demonstrates that the negative inotropic effect of R-PIA alone was not different between the mouse models. **C** Beating rate in beats per minute (bpm). *First *p* < 0.05 vs. Ctr

**Fig. 6:**
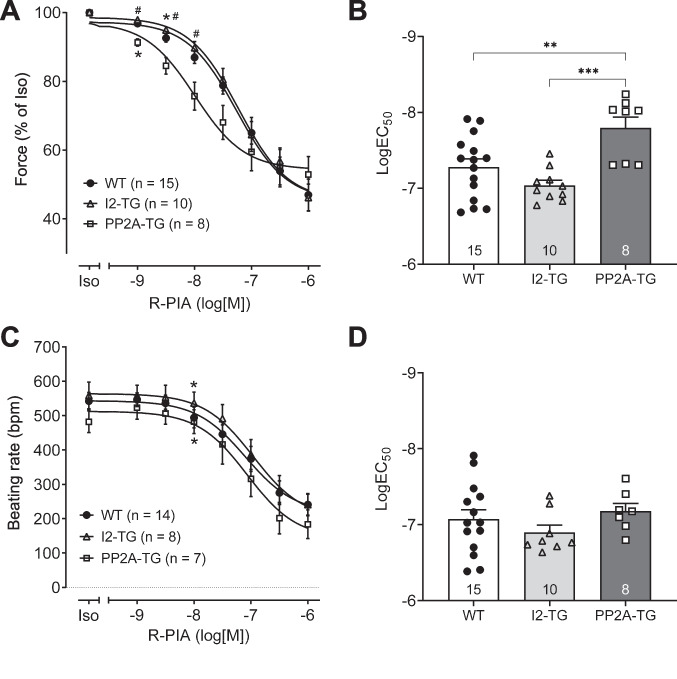
Summarized concentration–response curves for the negative inotropic and chronotropic effect of the adenosine receptor agonist R-PIA after pre-stimulation with 1 µM isoprenaline (Iso) in electrically driven (1 Hz) left atrial preparations (**A**, **B**) and in right atrial preparations (**C**, **D**), respectively, from PP2A-TG, I2-TG, and WT. Numbers in brackets give the number of experiments. Iso indicates the maximum response to isoprenaline and was used here as control for the R-PIA application. **A** Force of contraction in % of Iso. **B** The logarithm of the EC_50_ values demonstrates that the anti-adrenergic effect of R-PIA is enhanced in left atrial preparations from PP2A-TG compared to WT and I2-TG. Significant differences (*p* < 0.05) are indicated by brackets. **C** Beating rate in beats per minute (bpm). **D** The logarithm of the EC_50_ values demonstrates that the negative chronotropic effect of R-PIA after pre-stimulation with Iso was not different in right atrial preparations. *First *p* < 0.05 vs. Iso; ^#^*p* < 0.05 vs. PP2A-TG

For comparison, we analyzed the effects of the adenosine A_1_ receptor agonist R-PIA. R-PIA only exerts negative inotropic and chronotropic effects in atrial preparations from WT mice probably because a single receptor is involved (Neumann et al. 1999). For these experiments, only WT, PP2A-TG, and I2-TG mice were available. First, R-PIA was cumulatively applied (1 nM–1 µM) alone (that means without any pre-stimulation). The potency and efficiency of R-PIA for the negative inotropic effect in left atrial preparations or the negative chronotropic effect in right atrial preparations were not different between genotypes (Fig. 5). Next, atrial preparations were pre-stimulated with 1 µM isoprenaline before R-PIA was cumulatively applied. Now, in PP2A-TG left atrial preparations, the negative inotropic effect of R-PIA was more pronounced compared to WT and I2-TG (Fig. 6A, B). In isoprenaline-stimulated right atrial preparations, the negative chronotropic effect of R-PIA was not different between the genotypes (Fig. 6C, D).

### Protein phosphatase activity

**Fig. 7:**
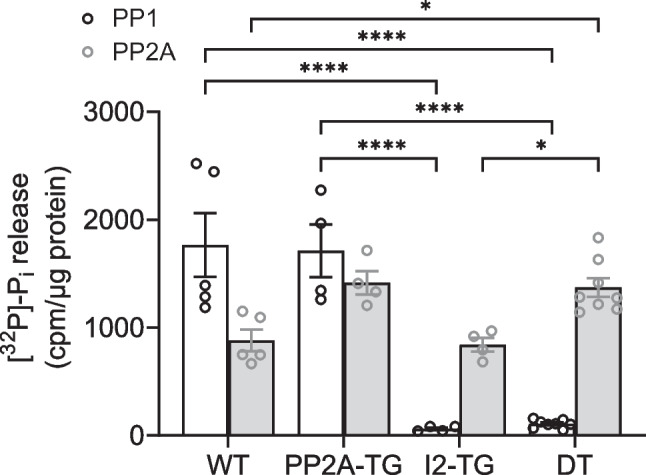
Protein phosphatase activity in whole heart homogenates. [^32^P]-phosphorylase *a* was used as a substrate, mainly metabolized by PP2A and PP1. The release of [^32^P]-Pi was measured as counts per microgram protein (cpm/µg protein). Of the protein phosphatase inhibitor okadaic acid, 10 nM was used to differentiate between PP1 and PP2A activity. Significant differences (*p* < 0.05) are indicated by brackets. *N* = 4–8

The activity of protein phosphatases was measured in whole heart homogenates of each genotype, using [^32^P]-phosphorylase *a* as substrate. By this substrate, mainly the activities of PP1 and PP2A are measured. To differentiate between PP1 and PP2A activity, 10 nM of the protein phosphatase inhibitor okadaic acid was used, because at the given low concentration of okadaic acid, PP2A activity is completely blocked whereas PP1 is still active (Gergs et al. 2004). Here, the relation of PP1 activity to PP2A activity was 2 to 1 in WT samples and nearly 1 to 1 in PP2A-TG hearts. In I2-TG and DT, PP1 activity was almost completely blocked, whereas the PP2A activity in I2-TG and DT was in the range of WT and PP2A-TG, respectively (Fig. 7).

## Discussion

All regions of the mammalian heart are parasympathetically innervated and the supraventricular tissues are more densely innervated than the ventricles. Vagal activation leads to stimulation of cardiac muscarinic receptors that modulate the activity of the sinus node, the atrioventricular conduction, and also the force of contraction (Dhein et al. 2001). The M_2_ muscarinic receptor is the predominant muscarinic receptor in the mammalian heart and its M_2_ receptors are abundantly expressed in the atrium (Lymperopoulos et al. 2021; Osipov et al. 2023). The M_2_ receptor signaling involves at least G_i/o_-mediated inhibition (or stimulation) of ACs, indirect (cAMP-dependent) or direct (cAMP-independent mediated by the α-subunit of G_o_) inhibition of I_f_, and activation of I_K.ACh_ (Dhein et al. 2001; Yatani et al. 1990). Moreover, there is a vast literature that cGMP concentrations are increased by acetylcholine (Dawson et al. 2008; Lang et al. 2007; Vente 2004). It was proposed that M_2_ receptors can inhibit as well as stimulate ACs depending on the AC isoform. AC isoforms 5 and 6 can be inhibited by M_2_ receptors via the α_i/o_ subunit of G_i/o_ and AC isoforms 4 and 7 can be stimulated by M_2_ receptors via the βγ subunit of G_i/o_ (Harvey and Belevych 2003).

Already more than 20 years ago, it was clear that other muscarinic receptors, different from the M_2_ receptor, exist in the mouse and human heart (overview in (Brodde and Michel 1999)). For instance, the presence of cardiac M_3_ muscarinic receptors has been demonstrated by functional and molecular biological studies, for example, in humans (Hellgren et al. 2000; Oberhauser et al. 2001), in rats (Bognar et al. 1990; Pönicke et al. 2003), or in mice (Hara et al. 2009; Kitazawa et al. 2009). Originally, no M_1_ receptor could be detected in the human heart (Hellgren et al. 2000), although the presence of the M_1_ receptor had been suggested based on functional data (Du et al. 1995). Meanwhile, the presence of G_q_-coupled M_1_ receptors that regulate the atrial I_K,ACh_ has been described in the human atrium (Heijman et al. 2018). While it is accepted that the M_2_ receptor exists in cardiomyocytes, the precise cellular localization of the M_3_ receptors, however, is not completely clear (Brodde and Michel 1999; Dhein et al. 2001; Kitazawa et al. 2009). Pönicke et al. (2003) found functional evidence for the presence of M_3_ receptors in isolated rat ventricular myocytes. In contrast, in mouse atria, the presence of M_3_ receptors was located in the endocardial endothelium (Hara et al. 2009). To summarize the current data, M_1_ and M_3_ receptors seem to be expressed in cardiomyocytes, but less abundant than M_2_ receptors and with species differences (M_1_ more in human hearts, M_3_ more in rodent hearts) (Lymperopoulos et al. 2021; Osipov et al. 2023). While many questions remain regarding the expression of muscarinic receptors in the heart, the expression of numerous protein phosphatases, including PP2A and PP1, in cardiomyocytes has been widely described (Gergs et al. 2019a; Heijman et al. 2013, 2017; Herzig and Neumann 2000; Neumann et al. 2021).

Nonetheless, muscarinic receptor signaling pathways are complex and there is still debate as to which receptor and which signaling pathway is associated with which function. Briefly summarized, the negative chronotropic and inotropic effects are commonly associated with the M_2_ receptor, and the positive inotropic effects in the mouse atrium are associated with the M_3_ receptor. Relatively little attention has been paid to date to the protein phosphatases as the regulator of muscarinic receptor signaling. But there is some evidence for the involvement of protein phosphatases. As mentioned in “Introduction,” in guinea pig perfused hearts and ventricular cardiomyocytes, ACh reduced via M_2_ receptors the isoprenaline-induced phosphorylation of PLB and TnI. These effects were independent of cAMP and PKA and were blocked by the protein phosphatase inhibitor okadaic acid (Gupta et al. 1994). In mouse and canine cardiomyocytes, M_2_ receptor stimulation led to an increase in the phosphorylation status of the ryanodine receptor 2 (RyR2) at serine-2808, while dephosphorylation of serine-2814 has been associated with activation of M_3_ receptors (Ho et al. 2016). The authors concluded that muscarinic receptor stimulation enhances the efficiency of cardiac sarcoplasmic reticulum calcium cycling by changes in the phosphorylation status of RyR2 at serine-2808 and serine-2814 (Ho et al. 2016). This mechanism may be influenced by protein phosphatases because, for example, PP2A decreases the phosphorylation status of RyR2 at serine-2808 (Hoehn et al. 2015). The phosphatase inhibitor sodium fluoride attenuated the negative inotropic effect of CCh in isoprenaline-stimulated guinea pig papillary muscles and these effects were reversed by the addition of deferoxamine that complexes and thus inactivates fluoride (Neumann et al. 1995; Neumann and Scholz 1998). In the canine ventricular myocardium, activation of a cantharidin-sensitive phosphatase was involved in the negative inotropic effects of CCh (Chu et al. 2003). A limitation of the study from Chu et al. (2003) was that only with higher concentrations of cantharidin (30 µM), an effect was notable. Based on the fact that the IC_50_ values of cantharidin for PP1 (IC_50_ = 1.7 µM) and PP2A (IC_50_ = 0.16 µM) are much lower, other protein phosphatases than PP1 and PP2A may be involved, for example, PP2B that is inhibited by a more than 500-fold higher concentration of cantharidin than PP1 (Herzig and Neumann 2000; Honkanen 1993; Neumann et al. 2021).

While the regulation of signal transduction through phosphorylation has been widely studied, little attention has been paid to the phosphorylation of the receptor itself. It is known that the activated M_2_ receptor is phosphorylated by G protein-coupled receptor kinases (GRK2/3) and that this phosphorylation regulates the formation of receptor-arrestin complexes initiating G protein–independent signaling (Drube et al. 2022; Li et al. 2015). Moreover, also the inactive receptor can be phosphorylated by GRK5/6. It is assumed that this phosphorylation in the absence of agonists mediates the basal receptor cycling and arrestin-mediated signaling (Li et al. 2015). As good as nothing is known about the involvement of specific protein phosphatases, for example, to reverse the GRK effects. If PP2A or PP1 are involved in GRK-mediated effects, we should see differences in our transgenic mouse models.

Motivated by these previous studies, we examined in the present work the atrial effects of muscarinic receptor stimulation in mice with either increased PP2A activity (Gergs et al. 2004) or with reduced PP1 activity (Brüchert et al. 2008; Dörner et al. 2021; Grote-Wessels et al. 2008; Krause et al. 2018) in the heart. Here, we have not measured the atrial protein phosphatase activity, but from our whole heart data presented here and from previous studies we know that in I2-TG hearts, not only PP1 activity is reduced but also, presumably due to counter-regulatory effects, the PP2A activity (Brüchert et al. 2008; Kirchhefer et al. 2005; Krause et al. 2018). Therefore, we crossbred the PP2A-TG with I2-TG mice, and in these double-transgenic animals, the cardiac activity of PP2A was shifted towards the activity found in monotransgenic PP2A-TG, whereas the PP1 activity remained as low as in the monotransgenic I2-TG. On the functional level, there seems to be no obvious interaction between PP2A and I2^PP1^, as the PP2A-induced cardiac hypertrophy was not affected by co-expression of I2^PP1^ (Brüchert et al. 2008). In contrast, in double transgenic PP1xI2 mice, the PP1-induced heart failure could be completely abolished by co-expression of I2^PP1^ (Brüchert et al. 2008). A more general inhibition of protein phosphatases in the heart, for example, with sodium fluoride or cantharidin, attenuated the negative inotropic effects of CCh or R-PIA (Chu et al. 2003; Neumann et al. 1995; Neumann and Scholz 1998). Therefore, we initially assumed that, if PP1 is involved, in I2-TG hearts, the effect of protein phosphatase inhibitors may be mimicked, at least in part, by the transgene. That is, the negative inotropic/chronotropic effect of CCh or R-PIA should be attenuated in I2-TG. On the other hand, if PP2A is involved, the effects of CCh and R-PIA may be potentiated in PP2A-TG or in DT. Taken together, our data suggest that neither PP2A nor PP1 are involved in the cardiac carbachol effects.

The negative inotropic effect of R-PIA seems to be potentiated in PP2A-TG, leading to the assumption that PP2A, at least in part, is involved in adenosine receptor signaling.

In CHO cells, it has been demonstrated that PP2A is involved in M_3_ receptor signaling. Via SET, an inhibitory subunit of PP2A, the M_3_ receptor dephosphorylation was decreased followed by a decreased G protein coupling (Simon et al. 2012). From these data, it could be concluded that a decreased activity of PP2A contributes to the inhibition of M_3_ receptor calcium signaling (Simon et al. 2012). In reverse, it could be assumed that an increased PP2A activity may positively contribute to M_3_ receptor signaling. Here, the missing effects of PP2A overexpression on the apparently M_3_ receptor–mediated positive inotropic effects led to the assumption that the PP2A is either not involved or the overexpressed catalytic subunit is not correctly targeted in PP2A-TG or the M_3_ receptor signaling in CHO cells is completely different compared to cardiomyocytes. On the other hand, if the M_3_ receptor is only located in the endocardial endothelium, as suspected by the authors, and the effects of the M_3_ receptor are mediated indirectly through the release of prostaglandins (Hara et al. 2009), the overexpressed PP2A may be ineffective because in our transgenic model, overexpression is restricted to the cardiomyocytes and does not occur in cardiac endothelial cells (Gergs et al. 2004).

Our current study has several limitations: firstly, in this work, we have not generated biochemical data like the phosphorylation level of regulatory proteins in the context of muscarinic receptor stimulation. In a future repetition of the experiments, it would be necessary to freeze clamp the sample time matched at the maximum negative inotropic effect as well as at the maximum positive inotropic effect. Secondly, we do not know exactly how the overexpressed catalytic subunit of PP2A is targeted in the transgenic mice because of missing data concerning the regulatory PP2A subunits also if the expression of important regulatory subunits seems to be unchanged in PP2A-TG compared to WT (Gergs et al. 2004). Thirdly, experiments with selective receptor antagonists to verify the involved receptor subtype are currently missing also because the development of subtype-selective small molecules has been challenging (Pham et al. 2023). However, experiments with supposedly M_1_ or M_3_ receptor–selective antagonists (Heijman et al. 2018; Liu et al. 2018) should be performed in a follow-up project. Moreover, the study is restricted to only one experimental method (measurement of the force of contraction of atrial preparations) and, therefore, a complementary method like isolated cardiomyocytes would be interesting to study in a follow-up project. Finally, other protein phosphatases like PP2B should be taken into account (Honkanen 1993).

In summary, we nonetheless speculate that protein phosphatases, perhaps different from PP1 or PP2A, are involved in the muscarinic receptor signaling. Therefore, further investigations should be carried out on this issue. Indeed, we have started to establish a transgenic model with overexpression of the PP2A inhibitor SET that may be helpful in future studies, but this is beyond the scope of the present work.

## Data Availability

The data of this study are available from the corresponding author upon reasonable request.
